# On the Aptness of Material Constitutive Models for Simulating Nano-Scratching Processes

**DOI:** 10.3390/ma17174208

**Published:** 2024-08-25

**Authors:** Hao Shen, Sivakumar Kulasegaram, Emmanuel Brousseau

**Affiliations:** School of Engineering, Cardiff University, Queen’s Buildings, The Parade, Cardiff CF24 3AA, UK; shenh4@cardiff.ac.uk (H.S.); brousseaue@cardiff.ac.uk (E.B.)

**Keywords:** nano-scratching, smooth particle hydrodynamics (SPH), Johnson–Cook model, elasto-plastic model

## Abstract

The simulation of nano-scratching on metallic substrates using smooth particle hydrodynamics (SPH) has been attempted by researchers in recent years. From a review of the existing SPH simulations of nano-scratching processes, it was found that mainly two different material constitutive models (i.e., the Johnson–Cook model and the elasto-plastic model) were employed to describe the material flow. In the majority of these investigations, the Johnson–Cook model was employed to characterise the stress flow of the material subjected to scratching. A natural question remains as to which material constitutive model is preferable for the SPH modelling of nano-scratching when quantitatively predicting the process outcomes. In this paper, a quantitative comparison of material responses during the nano-scratching of copper is reported when the process is simulated using SPH with two different constitutive material models, namely the Johnson–Cook and the elasto-plastic models. In particular, the simulated cutting and normal forces as well as the machined topography using both approaches are compared with the experimental work reported in the literature. The SPH-based simulation results in this paper are investigated based on the following three aspects: (a) cutting and normal forces with different material models and depths of the cut, (b) the effect of the cutting speed on forces and its dependence on adopted material models, and (c) the effect of adopted material models on the surface topography of machined nano-grooves. The SPH simulation results showed that using the Johnson–Cook material model, cutting and normal forces were closer to the experimental data compared to the results obtained with the elasto-plastic model. The results also showed that the cross-sectional profile of simulated nano-grooves using the Johnson–Cook model was closer to the experimental results. Overall, this paper shows that the selection of the Johnson–Cook model is preferable for the SPH modelling of the nano-scratching process.

## 1. Introduction

The development of novel micro- and nano-scale manufacturing technologies is a research area of high importance. Although vacuum and mask-based lithography techniques are employed in industry for the fabrication of semi-conductor devices and derived micro-electro-mechanical system (MEMS) components, they still have several limitations. In particular, these fabrication technologies generally rely on capital-intensive equipment while being restricted to the fabrication of planar features and constrained to a limited set of processed materials. In addition, there are also increased concerns over their environmental friendliness as they are energy- and resource-intensive and generate significant waste. New micro- and nano-scale manufacturing technologies, such as nano-scale mechanical machining, micro-electro discharge machining (micro-EDM), laser nano-machining, and focused ion beam (FIB) machining, have been developed by the research community to address these technological gaps. Among these technologies, nano-mechanical machining has the highest machining efficiency and can machine nano-scale products with intricate features.

Numerical simulations are extensively utilised for examining and predicting the responses of a range of materials in the context of nano-scratching or nano-machining processes. Many of these can be used to simulate a range of material responses for a variety of substrates including polymer, brittle materials, and metals. Molecular dynamics (MD) and the finite element method (FEM) are two most prevalent techniques implemented by researchers to simulate nano-scratching. Depending on the type of FEM approach followed, the technique may suffer from excessive mesh distortion, while MD is typically limited to simulations for several tens of nanometres and only up to several hundreds of picoseconds [1]. To overcome these issues, an alternative meshless method may be used [2]. Compared with FEM and MD, smoothed particle hydrodynamics (SPH), a mesh-free particle technique, is capable of handling large deformations in nano-scratching scenarios while avoiding the spatial and time limitations typical of MD. In line with the recent literature in which researchers reported the implementation of the SPH method to simulate the nano-scratching process, this paper further explores the capability of SPH in this context but with a specific focus on studying the effect of the adopted constitutive material model for such simulations.

In the published work focused on the application of SPH to simulate nano-scratching, also referred to as nano-machining in the literature, studies focused mainly on metallic materials. For instance, Zhao and co-workers [3] established an SPH model to study the influence of residual stress during repeated nano-scratching operations on oxygen-free high-conductivity copper (OFHC). Using the Johnson–Cook model to describe the material flow stress, these authors simulated chip formation, scratching forces, and residual stress. Geng et al. [4] also employed the SPH method with the Johnson–Cook model to simulate the orthogonal micro-scratching process on OFHC. In this case, the authors focused on investigating the effect of the frictional coefficient at the tool–specimen interface on the anticipated force of scratching and the form of the chips produced. Another SPH-based research work that specifically focused on the study of the machined surface profile and topography can be found in [5]. Zhong et al. [6] conducted SPH simulations to analyse the micro-scratching process with double-tip inclined angles on oxygen-free copper surfaces. This study revealed that increased tilt angles led to greater burr formation and affected the scratching forces, suggesting that optimising the tilt angle is crucial for enhancing the micro-scratching process. Guo et al. [7] studied the micro-machining of pure iron using SPH, with a specific emphasis on the role of the cutting edge radius in the chip formation process. The Johnson–Cook constitutive model was utilised in the SPH simulations. Guo et al. [8] implemented SPH-based nano-scratching simulations to validate theoretical analyses of the probe tip based nanochannel scratching process. The workpiece chosen was OFHC, and its flow stress was described with the Johnson–Cook model. Leroch et al. [9] used a total-Lagrangian SPH approach implemented in [10] to perform numerical simulations of nano-scratching on an elasto-viscoplastic substance, OFHC. Again, the Johnson–Cook model was deployed to serve as the material’s constitutive law. In addition to the SPH simulations of nano-scratching on metallic materials, researchers have also used various other models to simulate the nano-scratching process on different materials, such as potassium dihydrogen phosphate crystal (KDP) [11,12], monocrystal SiC [13,14], monocrystalline sapphire [15] and optical glass [16]. The material models employed in these studies were piecewise linear material model, Johnson–Holmquist ceramic (JHC) material model and JH-2 constitutive model within ANSYS/LS-DYNA, respectively.

As reported above, several papers [3,4,8,9] focused on simulating the nano-scratching process on copper specimens, specifically employing the Johnson–Cook model as the underlying constitutive framework. A common choice for the workpiece material across these studies was OFHC. However, the specific material parameters incorporated into the Johnson–Cook models were originally identified at the macro-scale. This common practice prompts an important question of whether the Johnson–Cook parameters established at the macro-scale can be directly and effectively applied to the simulations of nano-scale scratching processes. The resolution of this question is essential for the study of a range of material responses such as chip formation, processing forces, generated residual stress, and groove topography, as well as the effects of tool geometry during micro-/nano-machining for instance. In this context, the objective of the research reported here is to compare the influence of the adopted material model on the material response during nano-scratching by focusing on the predicted processing forces and resulting surface topography when simulating the process using SPH. More specifically, the comparison is made between the implementation of the Johnson–Cook and an elasto-plastic model. At the same time, the complementary objective of this work is to further investigate the feasibility of employing SPH modelling to simulate nano-scratching and nano-machining operations. The subsequent sections of this paper are structured as follows: Section 2 elucidates the foundational concepts of SPH and the SPH model of nano-scratching. Section 3 shows the simulation results using the SPH model with the Johnson–Cook and elasto-plastic models, with the specific focus on the cutting force, normal force and surface topography during nano-scratching. Finally, Section 4 presents the principal findings derived from this study.

## 2. Methods and Materials

### 2.1. SPH Method

SPH is a meshless Lagrangian computational method that can be implemented for simulating a range of engineering problems such as fluid flows, the fracture of brittle solids, metal forming and explosion phenomena. Its meshless nature particularly enables the efficient modelling of substantial material distortions, common in manufacturing processes like machining. Over the last decade, SPH has gained prominence as an alternative to FEM for machining process modelling. In SPH, the system’s dynamics are represented by discrete particles, each characterised by distinct material properties and adhering to the underlying conservation laws. These laws are often formulated as partial differential equations (PDEs), for which analytical solutions are typically elusive, mandating a numerical strategy. The process begins with the discretisation of the PDE domain, followed by the application of an approximation technique to estimate the field functions and their temporal derivatives at discrete points. This technique converts the PDEs into a set of ordinary differential equations (ODEs), which can then be solved using conventional finite difference method integration routines. In conclusion, the SPH method demonstrates its capability to solve complex PDEs by employing a systematic numerical approach, thereby solidifying its position as a robust tool in the realm of computational engineering.

In SPH, the computational domain is established by a collection of non-uniformly distributed particles, avoiding the need for pre-established connectivity. The approximation of field functions is accomplished through an integral representation technique, which endows the SPH method with essential mathematical stability. This integral-based process is known as kernel approximation within the SPH framework. The kernel function is subsequently refined by discretising it with particles, effectively replacing the integral with a summation of contributions from neighbouring particles within the kernel’s influence. The adaptability of SPH is highlighted by its iterative particle approximation at each time step, aligning with the changing local particle distribution. To maintain the integrity of the integration and ensure numerical stability, a sufficient count of particles must be engaged in the summation process. Upon approximation, particles are assigned a mass, thereby representing the physical material particles. The convergence of these strategies endows the SPH method with a mesh-free, adaptive, and stable approach to dynamic problems. Therefore, the SPH method stands out as a versatile tool for addressing problems with large deformations, ranging from conventional machining to the intricate challenges of micro- and nano-machining. Its robustness and adaptability make it a powerful candidate for tackling complex engineering scenarios.

### 2.2. SPH Modelling of Nano-Scratching

**Figure 1:**
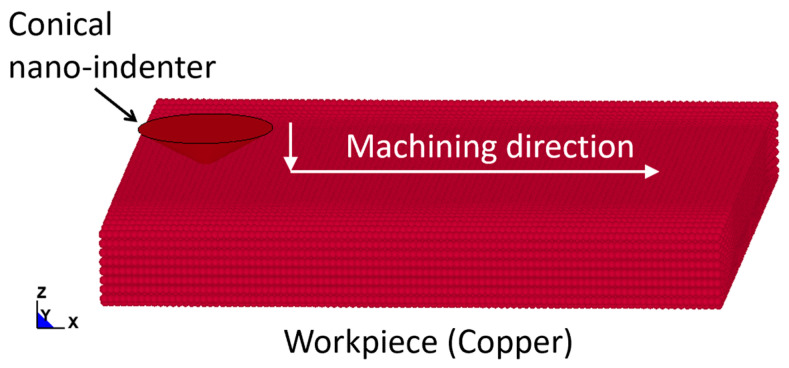
SPH simulation model for nano-scratching.

The SPH simulation model, as demonstrated in Figure 1, was specifically developed for this study to simulate the nano-scratching process. A diamond conical nano-indenter was selected for performing the nano-machining process on a copper sample. The indenter had the same negative rake angle of 60° and tip radius of 100 nm as those used in nano-scratching simulations and experiments in [17,18]. These two particular research studies were selected as benchmarks because they not only investigated the effects of nano-scratching conditions on material responses using SPH but also provide comprehensive experimental data to compare with. The dimension of the workpiece was 10 × 10 × 0.8 µm^3^. The software tools used to implement the SPH model of the process were ANSYS 2022 R2 and its LS-DYNA module. The indenter was designed, constructed, and meshed in the ANSYS environment first using a FE meshing scheme, and then imported into the LS-DYNA software. The workpiece was discretised solely using SPH particles with LS-DYNA.

In the nano-scratching model, the conical nano-indenter was modelled to move vertically into the material to achieve a specified depth of penetration, followed by a horizontal translation at this depth, parallel to the surface, to generate a scratch. For the purpose of performing a comparative analysis within this research, two distinct depths of penetration were selected: 100 nm and 150 nm. In order to improve the computational efficiency without sacrificing the accuracy of simulation results, the copper workpiece was discretised by SPH particles with varying densities. In particular, the discretisation of the model in the machining zone was refined to enhance precision, while the resolution was reduced near the bottom and side edges of the sample. Consequently, the size of the SPH particles spanned from 0.02 μm to 0.1 μm across the material. The SPH particle layers at the base of the copper specimen were fixed, restricting movement in all six degrees of freedom throughout the simulation.

For the majority of the simulations in this paper, a consistent scratching velocity of 10 m/s was applied, deviating only for the specific investigation of velocity’s influence, as elaborated in Section 3.2. There, three different scratch velocities, namely 100 m/s, 10 m/s, and 1 m/s, were investigated. While these values are higher than those typically encountered during nano-scratching experiments, especially with atomic force microscopes, they were deliberately chosen for two reasons. The first reason was to significantly reduce computational demands. Given the very small element and time step sizes in the implemented SPH model, employing conventional experimental velocities would have led to prohibitively long simulation times. Moreover, a review of the existing literature revealed that higher velocities, ranging from 0.1 m/s to 100 m/s, have been effectively utilised in SPH simulations for micro-/nano-scratching. Secondly, previous SPH simulation studies have shown that the resultant topographies of scratch-induced damage closely matched the experimental observations, and the forces predicted were also in reasonable alignment with the experimental data. This body of work supports the use of a higher velocity range to balance computational efficiency with the expected level of accuracy. Furthermore, the velocities selected for SPH simulations encompass a spectrum that aligns well with the acceptable range for SPH-based micro-/nano-scratching processes, as reported in the existing literature. Therefore, this selection of machining velocities ensures that this study not only advances the field but also remains grounded in established findings.

In this paper, the conducted simulation studies have utilised a specific contact model, “Contact_Automatic_Nodes_to_Surface”, also defined as contact model a5, within the LS-DYNA software environment. Some preliminary knowledge of the contact models in LS-DYNA is first briefly introduced before presenting the details of the adopted contact model. To enable flexibility in modelling contact, LS-DYNA presents a number of contact types. There are mainly three such contact types, namely the single-surface, node-to-surface, and surface-to-surface contacts. The single-surface contact type is suitable for addressing scenarios involving self-contact problems or large deformation problems where the pre-determination of the contact surface is difficult. The surface-to-surface contact is ideal for modelling contact where apparent sliding occurs on the contact interface. The node-to-surface contact is proposed for modelling contact where the surface of one part penetrates the surface of another. In particular, it is suitable for coupling mesh-based FEM and mesh-free SPH. LS-DYNA also defines a number of contact options, such as the General, Automatic, and Eroding contact options, for each contact type to control various aspects of the contact treatment.

In the adopted contact model a5 (“Contact_Automatic_Nodes_to_Surface”), the use of the “Nodes_to_Surface” contact type is to integrate the finite element-based nano-indenter with the SPH-particle-represented sample. This contact approach operates in a one-way manner, where only the indenter’s slave nodes are evaluated for potential penetration into the master segments of the workpiece. This one-way assessment is more efficient than the two-way method, which would necessitate an additional iteration to check for workpiece penetration into the indenter’s segments. The “Automatic” aspect of the contact option ensures that the mechanism is non-directional, adept at detecting penetration irrespective of the element’s orientation. This characteristic is particularly beneficial for simulating large deformation events, such as those occurring in nano-scratching. The efficiency, coupled with the model’s ability to handle complex deformation scenarios, makes “Contact_Automatic_Nodes_to_Surface” an optimal choice for the investigation of nano-scale material deformation in this study.

### 2.3. Materials

The diamond conical nano-indenter was modelled with a mass density of 3.515 g/cm^3^, an elastic modulus of 1140 GPa, and a Poisson’s ratio value of 0.29. As mentioned earlier, the flow stress of the copper workpiece material was described using two different approaches for comparison purpose, namely the Johnson–Cook and the elasto-plastic material models. The conventional Johnson–Cook model is often employed to describe the mechanical response of metallic materials under conditions characterised by large deformations and high strain rates. In particular, it can be expressed as

(1) $$\sigma =\left(A+B\varepsilon^{n}\right)\left[1+C\ln\left(\frac{\dot{\varepsilon }}{\dot{\varepsilon_{0}}}\right)\right]\left[1-{\left(\frac{T-T_{0}}{T_{m}-T_{0}}\right)}^{m}\right],$$

where *σ* symbolises the von Mises tensile stress, and *T* represents the temperature. The parameters *A, B, C, m, n,* ${\dot{\varepsilon }}_{0}$, *T*_0_, and *T_m_*, are defined as the yield strength, strength coefficient, strain rate parameter, temperature coefficient, strain hardening coefficient, a user-prescribed plastic strain rate, a reference temperature, and the melting temperature, respectively. *ε* denotes the equivalent plastic strain, and the rate at which this plastic strain changes is captured by the time derivative $\dot{\varepsilon }$. Material model 15 (“Mat_Johnson_Cook”) in ANSYS was used. The parametric values, as used in the current simulations, are presented in Table 1.

An elasto-plastic material model, “Mat_Plastic_Kinematic”, which is also defined as material model type 3 within ANSYS/LS-DYNA, was utilised to represent the plastic behaviour with isotropic and kinematic hardening. The parameter values used in these simulations are summarised in Table 2. It is highlighted that the yield stress and tangent modulus were chosen by different combinations of those found in the literature (i.e., yield stress 340–380 GPa and tangent modulus 38–48 GPa) to obtain the best machined surface in comparison with experimental results.

## 3. Results and Discussion

### 3.1. Forces Analysis during Nano-Scratching

**Figure 2:**
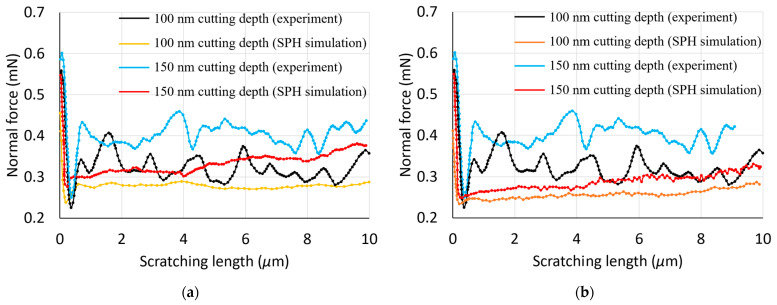
Experimental and simulated normal forces using the following: (**a**) a Johnson–Cook model; (**b**) an elasto-plastic model.

**Figure 3:**
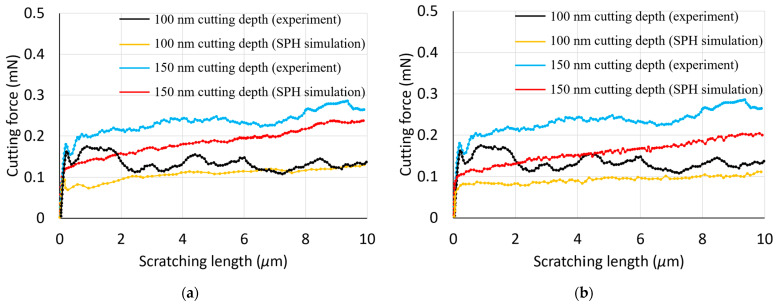
Experimental and simulated cutting forces using the following: (**a**) a Johnson–Cook model; (**b**) an elasto-plastic model.

In this section, the simulated cutting and normal forces are examined and compared with the experimental data from [17]. Figure 2 and Figure 3 display a comparative analysis of these forces for two cutting depths, 100 nm and 150 nm, across two material models. Notably, for both constitutive models, the simulated normal force exhibits a sharp initial decrease from a peak value, and then remains relatively stable during nano-scratching. This peak is associated with the maximum force upon completion of the initial nano-indentation step. Consistently, the normal force during nano-scratching is less than the maximum force observed during nano-indentation. The simulated cutting force shows a significant increase at the start of the scratching process, followed by a slight upward trend, yet it remains lower than the normal force throughout the simulations, aligning with the experimental results. With an increase in the depth of cut, both the normal and cutting forces increase, a pattern consistent with experimental findings.

While some discrepancies exist between the simulated and experimental forces, they are within a similar order of magnitude, indicating a reasonable agreement. This suggests that the SPH model could serve as a suitable initial approximation for predicting forces in nano-scratching scenarios. It is important to acknowledge, however, that the specific material properties of the copper substrate used in the referenced experimental work are not available, which limits the precision of a direct quantitative comparison with the reported experimental data. Despite this, the SPH model’s predictive capabilities are deemed reliable for preliminary assessments.

Figure 2 and Figure 3 illustrate a significant fluctuation in the experimental normal forces when compared with those predicted by SPH simulations. This is likely to be caused by inherent material defects and the possible influence of surface topography in practice. As reported in reference [18], the copper specimens underwent multiple preparation stages, including electro discharge machining, polishing, ultrasonic cleaning, and electroplating. These methods could introduce material defects, such as cracks, and induce secondary effects like hardening, along with unforeseen changes to the surface topography. The nano-scratching tests, conducted in a displacement-controlled setup, maintained a constant depth of cut. Considering the unpredictable distribution of defects and the complexity of realistic surface features, fluctuations in the measured normal force could be generated. In contrast, SPH simulations, with their refined discretisation in the contact zone and the absence of defects and topographical considerations, exhibit minimal and negligible normal force variations.

One possible reason behind the fact that simulated forces were always lower than the experimental ones may be that the implemented SPH model did not take into account the contact friction. Another likely contributing factor behind this observation should be associated with the lateral size effect (LSE). Kareer et al. [19] have specifically reported an increase in scratch hardness with a decreasing indenter penetration depth during nano-scratching on single-crystal copper. It should be noted that the LSE was not factored into the SPH model applied in this study, indicating a significant area for improvement in future research. By incorporating the LSE, subsequent studies can achieve a more accurate representation of nano-scratching processes.

With respect to the comparison of the simulated cutting and normal forces between the two material models, it can be seen that these are slightly smaller in the case of the elasto-plastic model when contrasted with those obtained using the Johnson–Cook model. This means that the discrepancy between simulated and experimental forces is larger when using the elasto-plastic constitutive model.

### 3.2. The Effect of Cutting Speed

**Figure 4:**
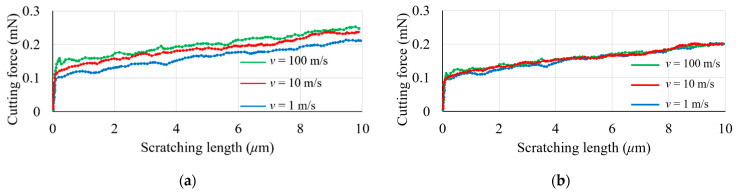
Influence of cutting velocity on cutting force at a 150 nm penetration depth using the following: (**a**) a Johnson–Cook model; (**b**) an elasto-plastic model.

**Figure 5:**
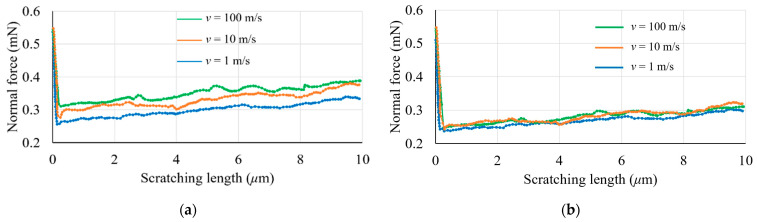
Influence of cutting velocity on normal force at a 150 nm penetration depth using the following: (**a**) a Johnson–Cook model; (**b**) an elasto-plastic model.

Figure 4 and Figure 5 depict the influence of the cutting velocity and adopted material model on the simulated forces. The velocities selected for the simulations, namely 100 m/s, 10 m/s, and 1 m/s, were applied to a penetration depth of 150 nm for both constitutive models. Although these scratching speeds are higher than those typically encountered in experimental tests, as discussed earlier, it can be said that the general trend is still reasonable when considering the Johnson–Cook material model. Specifically, as observed in Figure 4a and Figure 5a, the magnitudes of the cutting and normal forces become larger with an increase in cutting velocity. This is attributed to the sensitivity of the Johnson–Cook constitutive model to strain rates. These findings are also congruent with the simulation outcomes published by Guo and co-workers [8]. In contrast, this strain rate dependence is not observed, as expected, when using the elasto-plastic material model (see Figure 4b and Figure 5b).

Figure 4 also indicates that the cutting force experiences a steady rise as the scratching distance is increased. This gradual increase can likely be ascribed to the material’s pile-up and accumulation in the vicinity of the indenter during the nano-machining process. As further elucidated in Section 3.3, the simulated surface topography reveals a direct correlation between the machining distance and the volume of the material pile-up. A similar increasing trend is observed in the normal force, as shown in Figure 5. Due to the large negative rake angle (60°) of the conical nano-indenter, the material’s pile-up and accumulation in the vicinity of the indenter may also result in a progressive increase in the normal force during the nano-machining operation.

### 3.3. Machined Surface Profile during Nano-Scratching

**Figure 6:**
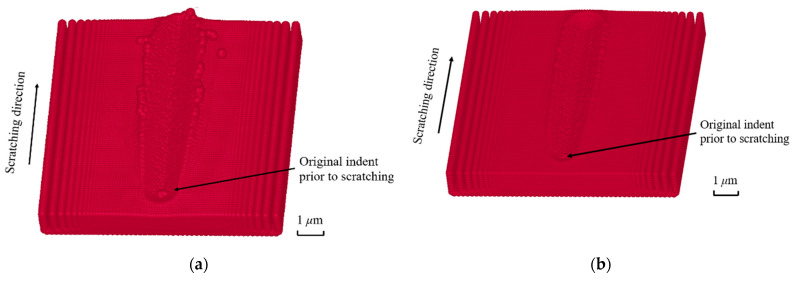
Simulated surface topography at a cutting depth of 150 nm using the following: (**a**) a Johnson–Cook model; (**b**) an elasto-plastic model.

This section investigates the workpiece surface resulting from nano-scratching. As shown in Figure 6, the simulated surface profiles are presented for both material models at a 150 nm cutting depth. In both cases, a groove is formed along the machining path, with material pile-ups generated on both sides of the cut groove. An additional observation in Figure 6a is the separation of several SPH particles from the surface. This finding, which departs from anticipated outcomes, could potentially be related to the high velocity applied during the simulation process. However, this phenomenon is not obvious in Figure 6b. This is because the values of yield stress and tangent modulus for the elasto-plastic model were chosen by different combinations of those found in the literature to obtain a machined surface with a minimum number of SPH particles which separated from the surface. Therefore, the separated particles in Figure 6a could also be related to the parametric values used in Table 1, which can be more fine-tuned in the future. It can also be seen from Figure 6 that no apparent fractures or chips are observed along the machining direction. Therefore, the ploughing mechanism was the dominant mode of surface generation for these nano-scratching simulations. This is also in line with the fact that for the corresponding simulation data reported in Figure 3 earlier, the values of the normal force were always higher than those of the cutting force.

**Figure 7:**
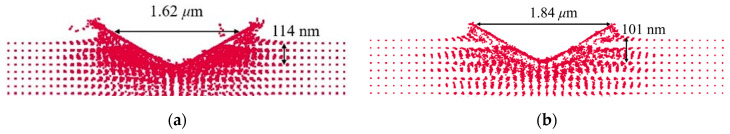
Cross-sectional profile of machined nano-groove at 150 nm depth of cut using the following: (**a**) a Johnson–Cook model; (**b**) an elasto-plastic model.

**Figure 8:**
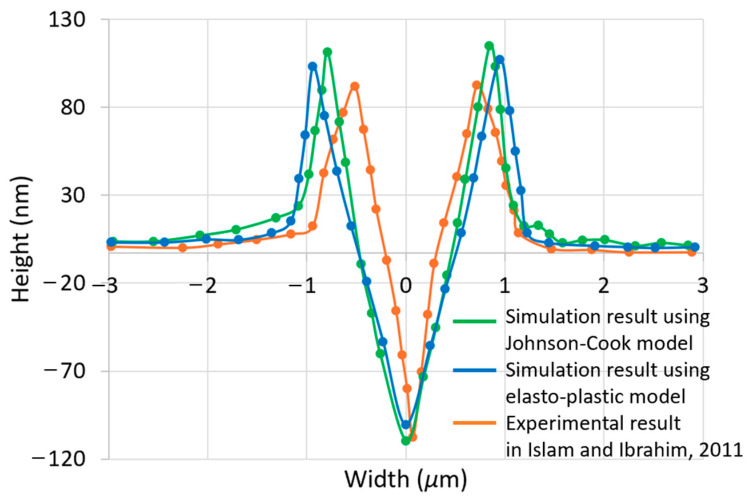
Comparison of cross-sectional profiles between simulated nano-grooves and experimental nano-groove [18].

Figure 7 shows the two-dimensional cross-sectional profiles of the scratched grooves which were simulated in Figure 6. These cross-sectional profiles can be visualised using the LS-Prepost software application. The nodal coordinates of interest were extracted from Figure 6 at a scratching length of 5 μm. This length corresponds to the mid-point of the total workpiece length and is considered to be an optimal observation point for obtaining a definitive representation of the simulated grooves. The conical indenter’s axial symmetry ensures that the geometry of the material accumulation on either side of the groove is significantly similar. For an enhanced perspective, Figure 8 offers a magnified view of the cross-sectional profiles initially presented in Figure 7. The experimental results found in Islam et al. [18] are also superimposed in Figure 8. A comparison reveals that the scratch width, as predicted by the Johnson–Cook model at a 5 μm scratch length, is 1.62 μm, slightly exceeding the empirical value of 1.24 μm. Furthermore, the model’s prediction of a scratch depth of 114 nm is in close proximity to the experimental measurement of 112 nm. The Johnson–Cook model’s predictions, when compared to the experimental data from [18], show a percentage discrepancy of 31% for the groove width and a minimal 2% for the groove depth. In the case of the elasto-plastic model, the scratch width and depth are 1.84 μm and 101 nm. This represents percentage differences of 48% and 10%, respectively, against the experimental groove dimensions. In addition, Table 3 presents the scratch depth and width for different material models and different scratch distances at intervals of 2 μm. The consideration of this additional data aims to mitigate the inaccuracies that arise from the random selection of a 5 μm scratching length. The Johnson–Cook model’s predictions, when compared to the experimental data, show a percentage discrepancy of 25% for the averaged groove width and a 2% for the averaged groove depth. Whereas these two quantities are 44% and 11% respectively, for the elasto-plastic model. Therefore, it can be said that the cross-sectional profile of the simulated nano-groove using the Johnson–Cook model is closer to the experimental results compared with that obtained using the elasto-plastic model.

The discrepancies between the SPH-predicted and experimental scratch width by using Johnson–Cook model could be related to the material models employed in SPH simulations. The workpiece materials used in SPH simulations and nano-scratching experiments may not be exactly the same. On the one hand, when investigating the Johnson–Cook material model, the OFHC with material parameters in Table 1 was employed. On the other hand, it is also important to note that the material properties for the specific copper substate used in the experimental work from [17,18] are not available. Hence, it is not feasible to perform an exact quantitative comparison with the experimental data reported here. Another possible reason for these discrepancies between the SPH-predicted and experimental scratch width could be related to the absence of the failure criterion in the employed Johnson–Cook material model. Therefore, the refinement of the adopted Johnson–Cook material model and the improvement of the contact model could be further investigated in order to potentially improve the predictability and reliability of the implemented SPH model of nano-scratching.

## 4. Conclusions

A nano-scratching model of copper was implemented using SPH to complement existing literature with a specific view to study the influence of the selected material constitutive model. This was achieved by considering the Johnson–Cook and an elasto-plastic model and by analysing their effect on the simulated forces and the surface topography of scratched nano-scale groove. The simulated data were also compared against experimental results available in the literature. The conclusions that can be drawn from this study are summarised as follows:

- As expected, the normal and cutting forces showed an increasing trend with the increasing scratching velocity when using the Johnson–Cook constitutive model, while the scratching speed had no influence on the simulated forces in the case of the elasto-plastic material model.
- The utilisation of the Johnson–Cook constitutive model in the SPH simulations led to cutting and normal forces which were higher than those obtained with the elasto-plastic model and closer to those observed experimentally.
- The cross-sectional profile of simulated nano-groove using the Johnson–Cook model was also closer to experimental results compared to that obtained when implementing the elasto-plastic model. For this reason, and also based on the analysis of the simulated forces, it can be said that the selection of the Johnson–Cook model is preferable for the SPH modelling of the nano-scratching process.

## Figures and Tables

**Table 1:** Parametric values of the Johnson–Cook material model for OFHC.

Parameters	Value	Reference
Density (*ρ*)	8.93 g/cm^3^	
Young’s modulus (*E*)	117 GPa	
Poisson’s ratio (*ν*)	0.30	
*A*	0.09 GPa	[8]
*B*	0.292 GPa	[8]
*C*	0.025	[8]
*m*	1.09	[8]
*n*	0.31	[8]
*T_m_*	1356 K	[8]
*T*_0_	293 K	[8]

**Table 2:** Parametric values of the elasto-plastic model for OFHC.

Parameters	Value
Density (*ρ*)	8.93 g/cm^3^
Young’s modulus (*E*)	117 GPa
Poisson’s ratio (*ν*)	0.30
Tangent modulus	43 GPa
Yield stress	360 GPa

**Table 3:** The scratching depth and width for different material models and scratching distances.

Scratch Distance (μm)	Johnson–Cook Model		Elasto-Plastic Model	
	Scratch Depth (nm)	Scratch width (μm)	Scratch Depth (nm)	Scratch Width (μm)
2	109	1.32	98	1.56
3	117	1.54	100	1.82
6	115	1.66	103	1.88
8	116	1.68	100	1.89
Averaged value	114	1.55	100	1.79
Experimental dimensions	112	1.24	112	1.24
Percentage difference	2%	25%	11%	44%

## Data Availability

The original contributions presented in the study are included in the article. Further inquiries can be directed to the corresponding author.
